# Health-related quality of life in Chinese workers: a systematic review and meta-analysis

**DOI:** 10.1186/s41256-021-00209-z

**Published:** 2021-08-13

**Authors:** By Ya Su, Meng-Shuang Liu, Pinnaduwage Vijitha De Silva, Truls Østbye, Ke-Zhi Jin

**Affiliations:** 1 Department of Occupational Health, School of Public Health, Fudan University, 138 Yixueyuan Road Box288#, Shanghai, 200032 P.R. China; 2 Key Laboratory of Public Health Safety, Ministry of Education, Shanghai, P.R. China; 3 Fudan Global Health Institute, Shanghai, P.R. China; 4 Department of Community Medicine, University of Ruhuna, Galle, Sri Lanka; 5 Duke Global Health Institute, Durham, NC USA

**Keywords:** Health-related quality of life, Occupational health, Systematic review, Meta-analysis, China

## Abstract

**Background:**

Studies of health-related quality of life among workers have generated varying results. The purpose of this study was to conduct a systematic review to synthesize the scores of health-related quality of life measured by the World Health Organization Quality of Life questionnaire among Chinese workers and compare the results across gender, age, occupation and region.

**Methods:**

Six databases including China National Knowledge Infrastructure, WanFang Data, China Science and Technology Journal Database, PubMed, Web of science and Scopus were searched for relevant publications in both English and Chinese from their inception to February 2021. Inclusion and exclusion criteria were established, and study and participant characters as well as health-related quality of life scores were extracted from included publications. Study quality was assessed by using the Crombie tool. The meta-analysis including individual publications used random-effects models. Subgroups analyses by gender, age, occupation and region were also conducted to explore the source of heterogeneity.

**Results:**

One hundred thirty-nine out of 1437 potential publications were included. The pooled mean scores of health-related quality of life were 14.1 for the physical domain (95%CI: 13.9–14.3), 13.7 for the psychological domain (95%CI: 13.5–13.8), 14.0 for the social relationship domain (95%CI: 13.8–14.2), 12.3 for the environment domain (95%CI: 12.1–12.5). No significant statistical difference was found between the different subgroups. Publication bias was present in the independence domain and the pooled scores were corrected to 15.0 (95%CI: 14.6–15.5) using the trim and fill method. Sensitivity analysis suggested that the results of the meta-analysis were stable. Region might be a source of heterogeneity. Workers in northeast China reported higher scores in the social relationship domain, and those in the central region reported lower scores in the environmental domain.

**Conclusions:**

Chinese workers reported lower scores in four health-related quality of life domains than the general population. Region might be a potential influencing factor for workers’ scores different, which needs further study. The pooled scores can served as benchmarks for workplace health promotion programes in Chinese workers and global occupational health studies.

## Background

Health-related quality of life (HRQOL) is defined by the World Health Organization (WHO) as “individuals’ perceptions of their position in life in the context of the culture and value systems in which they live and in relation to their goals, expectations, standards and concerns” [1]. According to this definition, HRQOL is not only related to an individual’s health status but also to their personal satisfaction. Therefore, HRQOL can vary greatly between China and other regions with different languages and culture. HRQOL instruments have been widely used in China since the 1980s. The Chinese versions of the World Health Organization Quality of Life (WHOQOL) questionnaire including WHOQOL-100 and WHOQOL-BREF were translated by Fang and his colleagues and were shown to have good reliability and validity in the Chinese population [2]. WHOQOL-BREF (26 items) is a simplified version based on WHOQOL-100 (100 items). The items from the two scales were grouped into 4 domains: physical health, psychological health, social relationship and environment as well as evaluate general HRQOL and general health. The scores in each domain have good comparability between the two scales: the Pearson correlation coefficient ranges from 0.89 (the social relationship domain) to 0.95 (the physiological domain) [3]. WHOQOL-100 includes two additional domains: independence and spirituality beliefs.

Occupational activities run through most people’s lives, and working conditions and environments have been recognized as important health determinants, i.e. key drivers of HRQOL. Different occupational groups may experience various and different health problems due to the nature of their jobs, with different performance in HRQOL. For example, high physical work demand and awkward static/repetitive working postures may contribute to higher incidence of musculoskeletal disorders [4, 5]; shift work is related to cardiovascular heart disease and mental disorders [6, 7]; and sedentary behavior is a risk factor for chronic diseases including obesity, diabetes, etc. In addition, male and female workers at different ages may have different types of job, e.g. nurses and teachers are mostly women, while blue-collar workers (such as construction workers and miners) are mostly young men. Therefore, it is important to assess HRQOL by gender, age and occupation to identify differences and group time trends with a view to providing group specific occupational health services. The influence of different geographical regions on the results also needs to be explored, taking into account differences in climate, lifestyle and subtle cultural differences.

Although individual studies have reported results based on WHOQOL in Chinese workers engaged in different occupations, there has been no other systematic review summarizing these findings. Therefore, the primary objective of this systematic review was to summarize the findings around six HRQOL domains in Chinese workers, so as to provide references for future studies and for health policy (Studies using either of the two versions of the questionnaires generated the scores for physical health, psychological health, social relationship and environment domain, while only those using WHOQOL-100 generated the scores for independence and spirituality beliefs domain). The second objective was to compare the results across gender, age groups and occupational groups in order to explore the characteristics of different subgroups and identify more vulnerable groups.

## Methods

The protocol for this systematic review with meta-analysis was registered in the International Prospective Register of Systematic Reviews (PROSPERO, Registration ID: CRD42020151775). The current review was reported by following the guidelines of Preferred Reporting Items for Systematic Reviews and Meta-Analysis (PRISMA) Statement [8]. Two reviewers (SU and LIU) independently searched and selected the publications. Any disagreement led to a consultation with the third reviewer (JIN) and resolved by reaching consensus.

### Data sources and search strategy

Potential publications were identified from six databases searched from their inception and up to February 2021: China National Knowledge Infrastructure (CNKI), WanFang Data (WF), China Science and Technology Journal Database (CQVIP), PubMed, Web of Science and Scopus. Of these databases, CNKI, WF and CQVIP mainly covered Chinese publications. Keywords, medical subject heading (MeSH) terms and free-text words were used as searching strings. The search strategy incorporated two principal components. The first related to the study population: Chinese workers with active employment and engaged in any specific industries. The second related to the health outcome, namely HRQOL evaluated by WHOQOL-BREF or WHOQOL-100. The exact search strategies are presented in Table 1.

**Table 1:** Search strategies in CNKI, WF, CQVIP, PubMed, Web of Science and Scopus

Database	Nation	Occupation group	Quality of life
CNKI	–	SU = (‘员工’ + ‘人员’ + ‘职员’ + ‘工人’ + ‘农民工’ + ‘务工’ + ‘工作者’ + ‘公司’ + ‘职业’)	TKA = (‘世界卫生组织生存质量’ + ‘WHOQOL’)
WF	–	主题:(“员工” OR “人员” OR “职员” OR “工人” OR “农民工” OR “务工” OR “工作者” OR “公司” OR “职业”)	摘要:(“世界卫生组织生存质量” OR “WHOQOL”)
CQVIP	–	M = (员工 OR 人员 OR 职员 OR 工人 OR 农民工 OR 务工 OR 工作者 OR 公司 OR 职业)	R = (世界卫生组织生存质量 OR WHOQOL)
PubMed	(China [ALL] OR Chinese [ALL])	(workplace[MH] OR occupations[MH] OR occupational groups[MH] OR work[MH] OR employ*[ALL] OR workplace[ALL] OR workplaces[ALL] OR occupation*[ALL] OR work*[ALL] OR profession*[ALL] OR labor[ALL] OR labour[ALL] OR job[ALL] OR jobs[ALL] OR personnel[ALL] OR personnels[ALL] OR staff[ALL] OR staffs[ALL] OR “green collar”[ALL] OR “pink collar”[ALL] OR “white collar”[ALL] OR “blue collar”[ALL] OR company[ALL] OR companies[ALL] OR corporation[ALL] OR corporations[ALL] OR enterprise[ALL] OR enterprises[ALL])	(“world health organization quality of life”[ALL] OR WHOQOL[ALL])
Web of Science	TS = (China OR Chinese)	TS = (employ* OR workplace$ OR occupation* OR work* OR profession* OR labo$r OR job$ OR personnel$ OR staff$ OR “green collar” OR “pink collar” OR “white collar” OR “blue collar” OR company OR companies OR corporation$ OR enterprise$)	TS = (“the world health organization quality of life” OR WHOQOL)
Scopus	TITLE-ABS-KEY (China OR Chinese)	TITLE-ABS-KEY (employ* OR workplace OR workplaces OR occupation* OR work* OR profession* OR labor OR labour OR job OR jobs OR personnel OR personnels OR staff OR staffs OR “green collar” OR “pink collar” OR “white collar” OR “blue collar” OR company OR companies OR corporation OR corporations OR enterprise OR enterprises)	ALL (“the world health organization quality of life” OR whoqol)

### Study eligibility

The inclusion criteria were as follows: (1) cross-sectional study, or cohort, intervention study reporting baseline data; (2) conducted in the Chinese mainland; (3) active occupational population with specific occupation; (4) HRQOL measured using WHOQOL-BREF or WHOQOL-100; (5) publications in Chinese or English until February, 2021. Publications were excluded if they: (1) did not report specific scores or standard deviations; (2) reported nonstandard data (incomparability data that were not calculated according to standard methods); (3) included workers with specific diseases; (4) repeated findings from other analyses that measured the same population at the same study period; (5) were special groups providing goods or services prohibited by local law (e.g. sex workers).

### Data extraction

The extracted data from the included publications contained: (1) study characteristics (author, published year, etc.); (2) participant characteristics (age, gender, occupation, region, response rates etc.); (3) health outcomes (sample sizes, average scores and standard deviation for different domain of HRQOL). Microsoft Excel 2016 was used for data management.

### Quality assessment

The methodological quality of each study was evaluated using a well-established quality appraisal tool recommended by Crombie [9]. The tool and its modified version have been used in many systematic reviews [10–12]. The tool consists of 7 items with responses “Yes (1 point)” or “No (0 point)”. Consequently, each study provided a score between 0 and 7. The scores were grouped into: ≤5 (low quality) and > 5 (high quality).

### Statistical analysis

To ensure comparability of data, scores on a 0–100 scale were transformed to a 0–20 scale following a procedure stated by the WHOQOL User Manual [13]. A meta-analysis was conducted for each domain of HRQOL to estimate the combined means and 95% confidence intervals. The test for heterogeneity among results and the selection of random effects model or fixed effects model were determined according to the I-squared statistics. Publication bias was assessed by Funnel plot, Egger’s test and Begg’s test. A *p*-value < 0.05 was considered statistically significant publication bias. The trim and fill method was further used to assess the influence of bias on the results. Influence analysis was conducted with each study deleted from the model to explore the stability of the results in the meta-analysis. Stata 15 was used for statistical analysis.

## Results

### Selection process

**Fig. 1:**
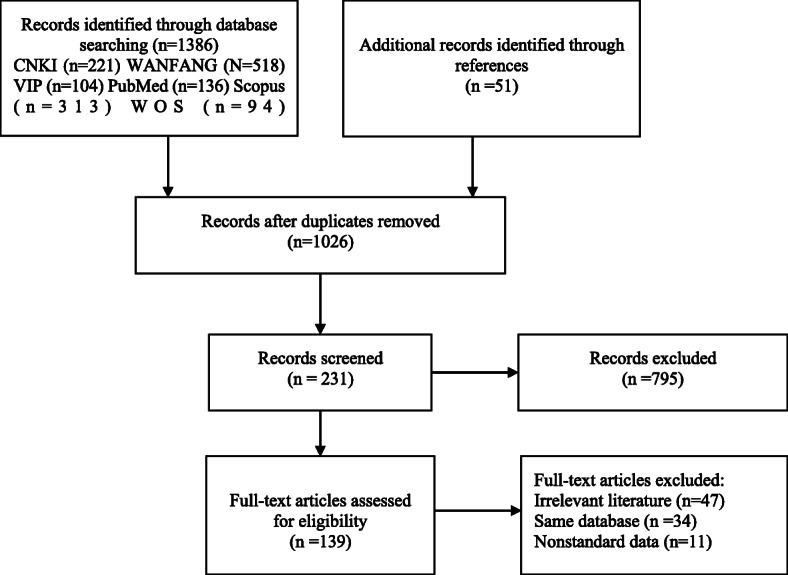
Flow diagram for selection of publications for quantitative synthesis, China

Figure 1 shows the flow diagram describing the study and publication selection process. A total of 1437 publications were initially identified from the databases or the related references, 1026 remained after removing duplicates electronically or manually. Next, 795 and 92 publications were removed by screening titles/abstracts and full text according to inclusion and exclusion criteria. Of the excluded full-text publications, 34 publications were found reported duplicated findings, and 11 publications were found reporting nonstandard data. The remaining 139 remained for quantitative synthesis.

### Publication characteristics and study quality

Table 2 describes the study and participant characteristics of each publication. The included publications reported a total of 98,144 workers engaged in construction, manufacturing, natural resource extraction, education, health and other working fields. Thirty-four publications only reported the numbers of participants in different age groups, so the mean age was estimated according to the mid-value of each age group. The mean age reported varied from 19.8 to 66.5. Female workers dominated the education and health care workforce, while male workers dominated in the military, mining, construction and manufacturing industries. The sample size reported in included publications ranged from 40 to 25,066, 60.4% (*n* = 84) had more than 300 participants. Twenty-one publications used the WHOQOL-100 questionnaire, whlie the remaining used the WHOQOL-BREF.

**Table 2:** Descriptive characteristics and quality assessment of the included publications

Author(year)	Occupation	Age (mean ± standard deviation, range)	Gender (%male)	Sample size (effective response rate)	Questionnaire	Region of work (province)	Quality assessment score
Huang et al. (2001) [14]	Nurses	31.2 ± 8.9, 18–55	0%	522 (94.9%)	100	Hubei	5
Liu et al. (2004) [15]	Medical staff	33.7 ± 9.1	37.3%	807 (89.7%)	100	Hunan	6
Wang et al. (2005) [16]	Military personnel	21.6 ± 3.7, 16–44	100%	612 (96.5%)	BREF	Inner Mongolia	6
Chen et al. (2005) [17]	Nurses	33.3 ± 8.7, 18–56	0%	1053 (90.0%)	100	Jiangsu	4
Li et al. (2005) [18]	Military convalescents	66.5 ± 9.7, 37–85	86.5%	244 (Unknown)	BREF	Guangdong	3
Jing et al. (2005) [19]	Oculists	33.3 ± 9.3^a^	32.2%	311 (94.2%)	BREF	Guangdong	6
Zhao et al. (2006) [20]	Military personnel	21.3 ± 3.0	100%	485 (99.0%)	BREF	Tibet	5
Geng et al. (2006) [21]	Armed polices	21.2 ± 3.1, 17–33	100%	1283 (100%)	BREF	Guangdong	4
Tang et al. (2006) [22]	Military personnel	20.8 ± 2.3, 17–33	100%	215 (Unknown)	BREF	Unknown	4
Tang et al. (2006) [23]	Hospital temporary workers	Unknown	Unknown	562 (93.7%)	100	Shenzhen	4
Yang et al. (2006) [24]	Middle school teachers	Unknown	18.4%	718 (89.4%)	BREF	Hebei	5
Liu et al. (2007) [25]	Nurses	29.9 ± 8.6^a^	Unknown	96 (96.0%)	100	Heilongjiang	3
Liu et al. (2007) [26]	Roadmen	29.8 ± 9.1	100%	376 (Unknown)	BREF	Hubei	4
Chen et al. (2007) [27]	Nurses	34.8 ± 9.2	Unknown	1648 (92.7%)	BREF	Shandong	4
Zhou et al. (2007) [28]	Middle SchoolTeachers	36.2 ± 8.0, 19–60	45.5%	622 (95.7%)	BREF	Hunan	6
Liu et al. (2007) [29]	Armed police forces	19.8 ± 1.9	100%	516 (97.4%)	BREF	Qinghai	6
Yang et al. (2008) [30]	Scientific research personnel	22–85	32.4%	272 (95.4%)	100	Beijing	5
Wang et al. (2008) [31]	Nurses	31.5 ± 4.9, 21–44	0%	189 (94.5%)	BREF	Guangdong	4
Tang et al. (2008) [32]	Military personnel	Unknown	Unknown	2581 (92.2%)	BREF	Unknown	5
Tang et al. (2008) [33]	Nurses	32.5 ± 8.5, 18–53	0%	574 (94.7%)	100	Guangdong	6
Du et al. (2008) [34]	Gym coaches	27.0 ± 5.6^a^	64.9%	97 (75.8%)	BREF	Shanghai, Jiangsu	5
Liu et al. (2008) [35]	Nurses	36.0, 18–60	0%	479 (95.8%)	BREF	Shandong	5
Yu et al. (2008) [36]	Coal workers	19–50	56.2%	505 (93.5%)	BREF	Shanxi	7
Zhang et al. (2008) [37]	Furniture maker	29.5 ± 8.6, 17–52	83.5%	85 (Unknown)	BREF	Beijing	5
Su et al. (2008) [38]	Middle SchoolTeachers	33.6 ± 7.5, 21–57	34.7%	759 (94.9%)	100	Shandong	6
Dong et al. (2008) [39]	Nurses	34.7 ± 8.3	Unknown	115 (76.7%)	100	Yunnan	3
Li et al. (2008) [40]	Doctors	39.7 ± 8.3	63.5%	200 (80.0%)	100	Chongqing	4
Liu et al. (2009) [41]	Reconstruction personnel after earthquake	39.5 ± 6.0	96.4%	112 (Unknown)	BREF	Sichuan	3
Tang et al. (2009) [42]	Military personnel	22.8 ± 3.8, 16–48	99.8%	2305 (95.8%)	BREF	Shanghai, Jiangsu, Jiangxi, Fujian	5
Gao et al. (2009) [43]	Nurses	32.9 ± 8.8, 20–52	Unknown	1018 (92.5%)	100	Yunnan	5
Wan et al. (2009) [44]	Nurses	31.9 ± 7.5^a^, 19–48	0%	499 (90.7%)	100	Hubei	5
Li et al. (2009) [45]	Nurses	33.4 ± 7.2^a^	0.4%	560 (94.0%)	BREF	Shaanxi	6
Zhou et al. (2009) [46]	Employees in finance, trading, technology, media, etc	29.7 ± 7.6, 19–59	35.9%	1001 (95.3%)	BREF	Shanghai	5
Zhang et al. (2009) [47]	Nurses	31.8 ± 8.1, 18–55	2.1%	610 (87.1%)	100	Xinjiang	7
Huang et al. (2009) [48]	Construction workers	Unknown	Unknown	1035 (Unknown)	BREF	Anhui	4
Huang et al. (2009) [49]	Train drivers	31.1 ± 6.9, 19–52	100%	230 (100%)	BREF	Guangdong	5
Ding et al. (2009) [50]	Construction workers	32.5 ± 10.0, 18–50	89.1%	101 (94.4%)	BREF	Shandong	5
Song et al. (2009) [51]	Journalists	Unknown	0%	117 (Unknown)	BREF	Unknown	3
Gu et al. (2009) [52]	Electronic enterprise workers	mainly 20–30 (64.9%)	31.6%	868 (86.8%)	100	Jiangsu	5
Song et al. (2009) [53]	Slaughterhouse workers	Unknown	Unknown	970 (64.3%)	BREF	Hebei	4
Liu et al. (2009) [54]	Medical staff	38.7 ± 9.9^a^	26.7%	664 (94.9%)	BREF	Liaoning	5
Wang et al. (2009) [55]	Education, scientific research, administrative management, medical technology and other workers	48.0 ± 5.5, 40–60	52.2%	1315 (84.3%)	BREF	Guizhou	6
Xing et al. (2010) [56]	Nurses	31.6 ± 6.9	5.1%	99 (82.5%)	BREF	Shandong	4
Bai et al. (2010) [57]	Civil servants	36.7 ± 8.4^a^, 20–60	51.3%	809 (95.2%)	BREF	Xinjiang	5
Wang et al. (2010) [58]	Medical staff	31.0 ± 9.1, 19–70	11.4%	404 (Unknown)	BREF	Beijing	4
Fu et al. (2010) [59]	Scientific research personnel	40.0, 27–56	72.7%	260 (Unknown)	BREF	Guangdong	3
Liu et al. (2010) [60]	Emergency nurses	28.9 ± 5.8, 20–58	6.1%	196 (93.3%)	BREF	Shandong	5
Zhang et al. (2010) [61]	Steel workers	38.1 ± 6.6, 19–51	92.7%	383 (95.8%)	BREF	Shanxi	5
Liu et al. (2010) [62]	Nurses	27.5 ± 6.2, 18–50	3.6%	1213 (93.3%)	100	Guangxi	5
Jiang et al. (2010) [63]	Construction, service, processing and manufacturing workers	24.6 ± 4.7^a^, 16–35	28.3%	265 (75.7%)	BREF	Fujian	5
Tang et al. (2010) [64]	Elementary and middle school teachers	22–59	44.4%	169 (92.9%)	100	Zhejiang	4
Yao et al. (2010) [65]	Medical college teachers	36.6, 24–59	33.6%	345 (95.8%)	BREF	Shanxi	5
Jin et al. (2011) [66]	Nurses	31.6 ± 9.1^a^, 19–53	0%	200 (Unknown)	100	Guangdong	3
Xu et al. (2011) [67]	Nurses	35.0 ± 8.0	Unknown	561 (93.5%)	BREF	Beijing	5
Lou et al. (2011) [68]	Medical staff	34.9 ± 9.1^a^	22.3%	452 (Unknown)	BREF	Shenzhen	5
Wang et al. (2011) [69]	Nurses	28.4, 19–45	0.3%	385 (96.7%)	BREF	Tianjin	5
Long et al. (2011) [70]	Doctors	23–60	57.0%	235 (78.3%)	BREF	Guangdong	4
Wei et al. (2011) [71]	Military personnel	21.2 ± 2.8, 18–34	100%	559 (98.4%)	BREF	Unknown	5
Ye et al. (2011) [72]	Military personnel	21.5 ± 2.9, 17–33	100%	554 (90.8%)	BREF	Yunnan	6
Wan et al. (2011) [73]	Policemen	Unknown	62.9%	70 (Unknown)	BREF	Yunnan	2
Xiong et al. (2011) [74]	Medical staff	33.4 ± 8.0	35.0%	331 (Unknown)	BREF	Hubei	5
Wang et al. (2011) [75]	Medical staff	37.0, 21–60	26.0%	672 (97.4%)	WHOQOL-BREF	Beijing	6
Zhang et al. (2011) [76]	Medical college teachers	37.0, 21–60	30.1%	249 (88.9%)	BREF	Anhui	5
Ma et al. (2012) [77]	Military personnel	37.6 ± 13.1^a^	100%	181 (90.5%)	BREF	Unknown	4
Ma et al. (2012) [78]	Peasant workers	26.8 ± 4.8	63.1%	756 (Unknown)	100	Hebei	3
Ban et al. (2012) [79]	Special education teachers	Unknown	35.9%	131 (87.3%)	BREF	Guizhou	4
Wang et al. (2012) [80]	Nurses	Unknown	Unknown	290 (96.7%)	100	Shenzhen	3
Hu et al. (2012) [81]	Enameled wire workers	32.5 ± 7.2, 19–55	74.3%	319 (Unknown)	BREF	Anhui	5
Xu et al. (2012) [82]	Nurses	31.0, 18–54	Unknown	287 (88.6%)	BREF	Guangdong	4
Zhang et al. (2012) [83]	Medical staff	> 40	21.5%	536 (97.1%)	BREF	Beijing	6
Liu et al. (2012) [84]	Electronic enterprise workers	34.9 ± 10.8^a^	10.0%	641 (98.6%)	BREF	Guangdong	4
Zhang et al. (2013) [85]	Service workers	24.3 ± 6.2^a^	0%	358 (Unknown)	BREF	Hebei	5
Xu et al. (2013) [86]	Nurses	34.2 ± 10.9^a^	2.0%	256 (88.6%)	BREF	Beijing	4
Wang et al. (2013) [87]	Employees in public places	30.1 ± 8.0, 19–57	27.5%	200 (Unknown)	BREF	Anhui	4
Hu et al. (2013) [88]	Civil servants	33.6 ± 10.5	55.4%	514 (93.5%)	BREF	Chongqing	5
Tan et al. (2013) [89]	Medical staff	39.8 ± 11.1^a^	Unknown	273 (Unknown)	BREF	Guangdong	2
Shan et al. (2013) [90]	Medical staff	37.0 ± 8.6	54.9%	82 (82.0%)	BREF	Zhejiang	4
Wu et al. (2013) [91]	Doctors	34.9 ± 5.9, 21–48	38.1%	291 (89.8%)	BREF	Fujian	4
Xing et al. (2013) [92]	Manufacturing, food and domestic service, retail sector, construction industry, transportation and other workers	39.9 ± 12.2^a^, 20–65	48.4%	1869 (93.5%)	BREF	Zhejiang	6
Yu et al. (2013) [93]	Nurses	24.4 ± 3.5	10.5%	468 (78.0%)	BREF	Hunan	6
Fu et al. (2013) [94]	Nurses	27.5 ± 5.0, 19–50	0%	310 (91.2%)	100	Henan	4
Zhang et al. (2013) [95]	Nurses	Unknown	47.1%	374 (93.5%)	BREF	Shandong	6
Wu et al. (2013) [96]	Foundry enterprise workers	26.4 ± 2.8, 22–39	82.4%	901 (91.5%)	BREF	Anhui	6
Geng et al. (2013) [97]	Nurses	43.8 ± 9.1^a^	0%	793 (88.1%)	BREF	Beijing and Tianjin	5
Lin et al. (2014) [98]	Medical staff	31.2 ± 8.0, 18–57	0%	315 (95.5%)	BREF	Fujian	6
He et al. (2014) [99]	Peasant workers engaged in non-agricultural production work	39.2 ± 8.8^a^	70.6%	436 (86.7%)	BREF	Unknown	4
Li et al. (2014) [100]	Nurses	18–30	0%	450 (88.2%)	BREF	Henan	6
Guo et al. (2014) [101]	Network, communications, pharmaceutical, banking and other industries staff; mining workers; construction workers	28.6 ± 4.9, 20–46	Unknown	1165 (Unknown)	BREF	Beijing	3
Li et al. (2014) [102]	Nurses	34.3 ± 9.3	0%	356 (96.2%)	BREF	Heilongjiang	6
Lao et al. (2014) [103]	Doctors	29.5 ± 4.0, 19–50	77.4%	1064 (62.6%)	BREF	Hunan	6
Wang et al. (2014) [104]	Military personnel	34.5 ± 6.8	100%	445 (Unknown)	BREF	Unknown	4
Zhang et al. (2014) [105]	Community nurses	20.7 ± 3.0	8.2%	232 (96.3%)	BREF	Jiangsu	5
Yang et al. (2014) [106]	Kindergarten teachers	33.2 ± 5.3, 18–60	14.6%	403 (91.6%)	BREF	Guizhou	6
Han et al. (2014) [107]	Nurses	28.0 ± 8.0, 16–50	0%	102 (92.7%)	BREF	Shanghai	4
Wu et al. (2014) [108]	Nurses	28.4 ± 5.5, 22–48	0%	215 (97.7%)	BREF	Henan	4
Zhang et al. (2015) [109]	Nurses	28.9 ± 7.8, 20–48	36.5%	181 (97.8%)	BREF	Shandong	5
Yang et al. (2015) [110]	HIV / AIDS prevention and control personnel	28.8, 23–48	31.6%	250 (100%)	BREF	Guangxi	5
Guan et al. (2015) [111]	HIV / AIDS prevention and control personnel	32.5 ± 8.4, 19–60	46.0%	250 (100%)	BREF	Heilongjiang	5
Li et al. (2015) [112]	Medical staff	39.7 ± 8.6, 21–63	2.6%	76 (Unknown)	BREF	Henan	4
Jiang et al. (2015) [113]	Railway construction workers	29.1 ± 10.9, 22–45	98.3%	950 (94.0%)	BREF	Shanxi	6
Miao et al. (2015) [114]	Nurses	29.4 ± 11.6, 24–44	Unknown	268 (95.7%)	BREF	Heilongjiang	4
Tang et al. (2015) [115]	Doctors	39.9 ± 11.3^a^, 15–65	51.7%	576 (91.4%)	BREF	Guangdong	6
Kang et al. (2015) [116]	Medical rescuers	31.4 ± 6.9^a^	33.7%	303 (89.6%)	BREF	Gansu	7
Yan et al. (2015) [117]	Doctors	40.2 ± 8.5	90.0%	60 (96.8%)	BREF	Guangdong	4
Pan et al. (2015) [118]	Nurses	32.6 ± 7.3	11.8%	152 (95.0%)	BREF	Guangdong	4
Chen et al. (2016) [119]	Sanitation workers	32.8 ± 12.9^a^	43.8%	121 (63.0%)	BREF	Ningxia	4
Dai et al. (2016) [120]	Civil servants	32.7 ± 8.6, 19–54	57.5%	708 (79.8%)	BREF	Jiangsu	5
Hu et al. (2016) [121]	Workers in a chemical enterprise	51.1 ± 9.7^a^, 30–70	71.4%	538 (90.7%)	BREF	Anhui	6
Yang et al. (2016) [122]	Workers in nonferrous metal ore concentrator, smelting enterprise, lead acid battery enterprise	35.8 ± 9.5, 21–59	0%	652 (97.3%)	BREF	Guangdong	5
Zhao et al. (2016) [123]	Military personnel	40.9 ± 10.1^a^, 18–59	87.5%	616 (94.8%)	BREF	Unknown	5
Tang et al. (2017) [124]	Nurses	39.9 ± 9.1^a^, 22–54	Unknown	40 (Unknown)	100	Liaoning	2
Zhang et al. (2017) [125]	Medical staff	22.6 ± 4.9, 17–47	37.7%	239 (95.2%)	BREF	Tibet	5
Lai et al. (2017) [126]	Nurses	32.1 ± 9.0^a^	0%	100 (Unknown)	BREF	Shenzhen	3
Zhao et al. (2017) [127]	Medical staff	35.5 ± 5.1, 20–50	Unknown	406 (81.2%)	BREF	Shaanixi	5
Xiao et al. (2017) [128]	Seafarers	Unknown	100%	917 (98.7%)	BREF	Jiangsu	6
Su et al. (2017) [129]	Armed polices	33.5 ± 9.6	100%	1327 (95.8%)	BREF	Shanxi	6
Liu et al. (2017) [130]	Doctors	21.0 ± 1.4, 17–34	68.1%	276 (92.3%)	BREF	Hubei	4
Zhang et al. (2017) [131]	Coal workers	45.9 ± 11.1^a^	63.7%	881 (97.9%)	BREF	Shanxi	7
Yi et al. (2018) [132]	Coal miners	37.7 ± 8.5, 18–65	Unknown	263 (87.7%)	BREF	Henan	4
Zeng et al. (2018) [133]	Military personnel	38.7 ± 7.9	100%	154 (96.3%)	BREF	Unknown	4
Yang et al. (2018) [134]	Service workers	24.9 ± 3.8	26.6%	139 (Unknown)	BREF	Yunnan	3
Lu et al. (2018) [135]	Migrant workers in Construction industry, catering industry, etc	31.1 ± 9.7^a^, 16–56	55.4%	267 (95.7%)	BREF	Tianjin	4
Zhao et al. (2018) [136]	Nurses	25.9 ± 4.7^a^, 18–36	Unknown	282 (95.6%)	BREF	Hebei	4
Xue et al. (2018) [137]	Nurses	36.8 ± 9.7^a^	0%	400 (87.0%)	BREF	Jiangsu	6
Song et al. (2018) [138]	Medical staff	32.8 ± 12.9^a^	23.2%	2274 (91.0%)	BREF	Beijing	5
Yang et al. (2018) [139]	University teachers	36.0, 20–70	47.0%	25,066 (78.3%)	BREF	Unknown	7
Yu et al. (2019) [140]	Nurses and other medical staffs	37.2 ± 7.8^a^, 24–65	29.6%	230 (Unknown)	BREF	Fujian	3
He et al. (2019) [141]	Nurses and other medical staffs	38.0 ± 3.2, 30–46	18.5%	200 (Unknown)	BREF	Hebei	3
Song et al. (2019) [142]	Nurses	31.1 ± 3.4, 22–45	0%	558 (93.0%)	BREF	Liaoning	5
Ma et al. (2019) [143]	Coal workers	Unknown	84.2%	3090 (71.2%)	BREF	Shanxi	6
Asante et al. (2019) [144]	Primary healthcare workers	51.7 ± 12.6^a^, 20–65	50.9%	873 (87.3%)	BREF	Guangdong	6
Zhu et al. (2019) [145]	Nurses	32.4 ± 6.9^a^	100%	315 (95.5%)	BREF	Shandong	6
Wu et al. (2020) [146]	Fishermen	27.9 ± 5.6^a^	99.4%	507 (Unknown)	BREF	Hainan	5
Zeng et al. (2020) [147]	Nurses	36.9 ± 11.3, 16–66	80.5%	1449 (68.2%)	BREF	Unknown	5
Liu et al. (2020) [148]	Nurses	32.6 ± 8.8	9.3%	75 (Unknown)	BREF	Tianjin	3
Luo et al. (2020) [149]	White-collar workers	29.1 ± 6.2, 21–40	28.0%	410 (Unknown)	BREF	Zhejiang	5
Wang et al. (2020) [150]	Military personnel	34.3 ± 9.2	100%	146 (97.3%)	BREF	Unknown	4
Wei et al. (2020) [151]	Pediatricians and Pediatric Nurses	24.3 ± 4.0	11.8%	355 (93.4%)	BREF	Henan	6
Chen et al. (2021) [152]	Radiation workers	32.2 ± 8.3^a^	69.9%	449 (89.8%)	BREF	Guangdong	5

^a^Represents that mean age and standard deviation of this publication was estimated by age frequency

The study quality assessment of those publications can also be seen in Table 2. The average score was 4.7, ranging from 3 to 7. 74.8% (*n* = 104) of publications were rated as having low study quality. The variation in scores mainly reflected in the items “appropriateness of design to meet the aims” and “clearly stated aims and likelihood of reliable and valid measurements”. Only 9 publications explicitly stated that random sampling or the whole population was used, and only 54.7% (*n* = 76) reported reliability or validity of the questionnaires used in the investigation.

### Meta-analysis

**Fig. 2:**
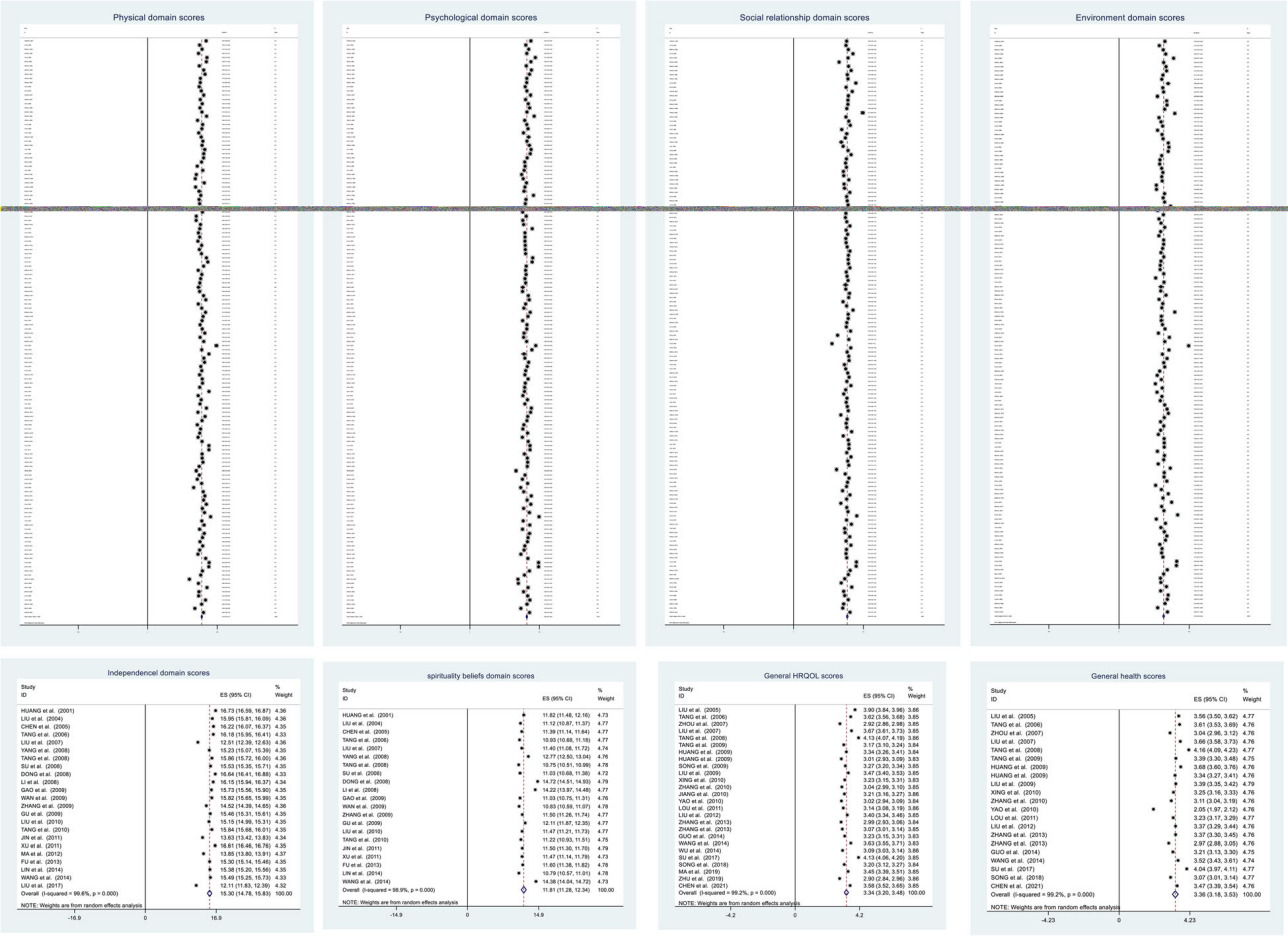
Forest plot for scores in the physical, psychological, social relationship, environment, independence, and spirituality beliefs domains, general HRQOL and general health, China, inception-2021. Note: all analyses were based on a random-effects model

The scores in the physical (*n* = 138), psychological (*n* = 138), social relationship (*n* = 137), environment (*n* = 136), independence(*n* = 23) and spirituality beliefs (*n* = 21) domains varied from 10.9–18.0, 11.1–16.6, 10.0–18.1, 10.0–19.2, 12.1–16.7, and 10.8–14.7, respectively. The heterogeneity test showed significant differences among the results of included publications, I^2^ > 98%, *P* < 0.001. Therefore, the random effects model was used for data synthesis. The estimated mean scores were 14.1 for the physical domain (95%CI: 13.9–14.3), 13.7 for the psychological domain (95%CI: 13.5–13.8), 14.0 for the social relationship domain (95%CI: 13.8–14.2), 12.3 for the environment domain (95%CI: 12.1–12.5), 15.3 for the independence domain (95%CI: 14.8–15.8), and 11.8 for the spirituality beliefs domain (95%CI: 11.30–12.3). Besides, 26 publications reported the general HRQOL and 21 reported general health, and the pooled scores were 3.3 (95%CI: 3.2–3.5), 3.2(3.2–3.5). The forest plots are shown in Fig. 2.

The analysis included publications from 2001 to 2021. HRQOL scores in the six domains each year were similar and showed no trend over time (*P >* 0.05). The maximum differences in the mean score for the physical, psychological, social relationship, and environment domain from year to year were 1.8, 1.4, 1.1, and 2.3, respectively.

### Publication bias assessment and sensitivity analysis

**Fig. 3:**
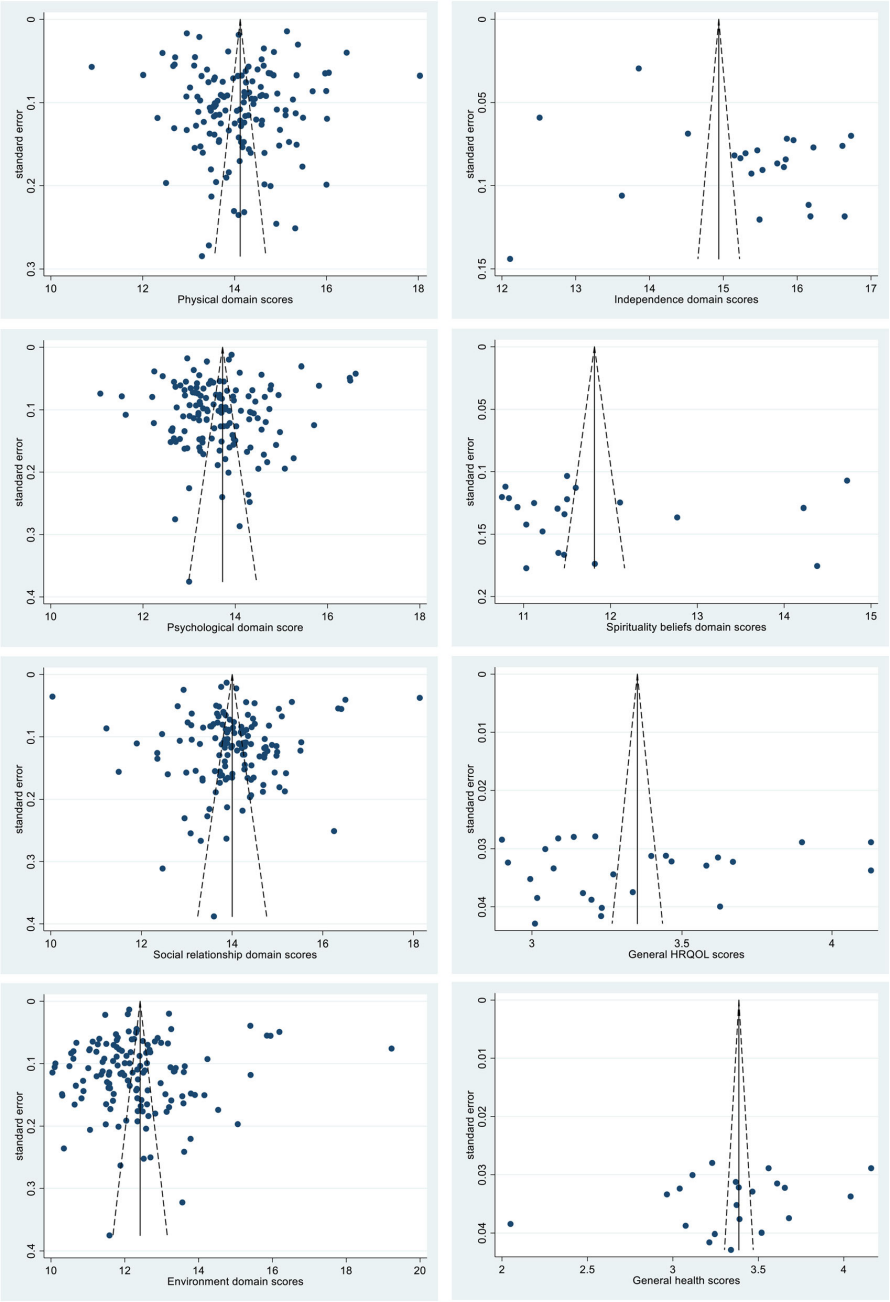
Funnel plots for selected indicators of HRQOL, China, inception-2021

Visual inspection of the funnel plot (Fig. 3), Egger’s test and Begg’s test did not suggest publication bias in the meta-analysis of the physical, psychological, social relationship environment and spirituality beliefs domains (*P* > 0.05). However, Egger’s test suggested potential publication bias in the independence domain (*P* = 0.011), while Begg’s test did not (*P* = 0.853). Therefore, the trim and fill method was also applied, and it indicated that if 4 estimated missing publications were added, then the pooled score of the independence domain would change to 15.0 (95%CI: 14.6–15.5). The sensitivity analysis demonstrated that when removing any one publication, the pooled scores were not altered significantly, with the overall changes differing only by 0.03 (0.2%), 0.02(0.2%), 0.03(0.2%), 0.05(0.4%), 0.14(0.9%), and 0.15(1.2%) in the physical, psychological, social relationship, environment, independence and spirituality beliefs domain respectively.

### Subgroup analysis

The data in the included publications were further analyzed by gender, age, occupation and region. Publications presenting multiple subgroups were included in the subgroup meta-analysis if the scores were reported for the respective subgroups. The gender characters were categorized into three based on gender dominance: male-dominated (> 80%, *n* = 49), female-dominated (> 80%, *n* = 60), and mixed (*n* = 34). 17 publications did not report participants’ gender and 22 publications reported age-specific results. The mean age of participants was divided into 3 categories: 19.8–29.9 (*n* = 38), 30.0–39.9 (*n* = 72), and 40.0–66.5 (*n* = 8). Twenty publications did not report the mean age or sample size of each age group. The occupations were grouped into three: workers in mining, construction and manufacturing were classified as blue-collar workers (*n* = 51); education, logistics and company staff as office workers (*n* = 20); and doctors, nurses and medical rescue workers as health care workers (*n* = 70). In addition, 2 publications reported occupation-specific results. Besides, we divided China into 8 geographical regions: central (*n* = 16), north (*n* = 26), east (*n* = 33), south (*n* = 25), southwest (*n* = 13), northeast (*n* = 6), northwest (*n* = 7). Twelve publications did not report the study region.

The pooled mean scores and 95% confidence intervals for the four HRQOL domains for each subgroup are presented in Table 3. No significant differences were found among different gender, age, and occupation groups, so these factors could not be regarded as sources of heterogeneity. The differences among regions were mainly reflected in social relationships and environmental domain. The pooled score of social relationship domain in northeast China was higher than that of other regions, while the pooled score of environmental domain in Central china was lower than that of other regions.

**Table 3:** Subgroup analyses: effect size by study characteristics

Subgroup	Physical domain	Psychological domain	Social relationship domain	Environmental domain
**Gender**				
Male-dominated	14.0 (13.7–14.3)	13.6 (13.3–13.8)	13.8 (13.5–14.0)	12.4 (12.0–12.8)
Female-dominated	14.2 (13.8–14.5)	13.6 (13.3–14.0)	13.8 (13.4–14.3)	12.2 (11.7–12.7)
Mixed	14.1 (13.8–14.5)	13.6 (13.5–13.8)	13.9 (13.7–14.1)	12.3 (12.1–12.5)
**Age**				
19.8–29.9	14.1 (13.7–14.5)	13.7 (13.4–14.1)	14.1 (13.7–14.4)	12.3 (11.9–12.8)
30.0–39.9	14.2 (13.9–14.5)	13.7 (13.5–13.9)	13.9 (13.6–14.2)	12.4 (12.0–12.7)
40.0–66.5	13.9 (13.3–14.6)	13.4 (12.8–13.8)	14.0 (13.8–14.2)	12.3 (12.1–12.5)
**Occupation**				
Manual workers	14.3 (13.9–14.5)	13.8 (13.5–14.1)	14.2 (13.7–14.6)	12.3 (11.9–12.7)
Office workers	14.0 (13.8–14.3)	13.5 (13.3–13.8)	13.9 (13.7–14.2)	12.3 (12.0–12.6)
Health care workers	14.2 (13.7–14.7)	13.7 (13.5–13.8)	14.0 (13.8–14.2)	12.4 (11.8–13.0)
**Region**				
Central China	14.1 (13.5–14.7)	13.4 (13.1–13.8)	13.7 (13.5–14.0)	11.7 (11.3–12.1)
North China	14.1 (13.7–14.5)	13.7 (13.2–14.2)	14.2 (13.6–14.7)	12.3 (11.7–12.9)
East China	14.1 (13.8–14.4)	13.6 (13.3–13.9)	14.2 (13.9–14.4)	12.1 (11.7–12.5)
South China	14.1 (13.6–14.6)	13.7 (13.3–14.1)	13.9 (13.6–14.3)	12.6 (12.2–13.0)
Southwest China	14.4 (13.6–15.3)	13.8 (13.1–14.4)	13.3 (12.1–14.5)	13.0 (11.2–14.8)
Northeast China	14.1 (13.7–14.4)	13.6 (13.2–14.0)	14.7 (14.2–15.2)	12.9 (12.0–13.8)
Northwest	13.6 (13.4–13.8)	13.6 (13.4–13.8)	14.0 (13.8–14.2)	11.8 (11.2–12.4)

## Discussion

Meta-analysis is increasingly being utilized in the health field. Meta-analysis of studies without control groups, with continuous outcome variables, only provide data for specific populations. Although investigations of HRQOL in different occupational groups have been conducted in several regions of China, there is great variation by gender, age, occupation and sample size, and these discrete results may therefore be difficult to compare between studies or use as an index to reflect changes after implantation of policy or health program. Therefore, we systematically reviewed the literature on occupational quality of life and attempted to estimate combined scores.

A total of 139 publications were included in meta-analysis, of which 136 were based on cross sectional studies, and 3 on intervention studies. Similar to most studies [153], the results of our meta-analysis on Chinese occupational groups showed that higher scores were found in the physical and social relationship domain than in the psychological domain, followed by the environment domain. Compared to the results from two large surveys conducted in China, a survey of 777 healthy participants [2] and a survey of 83,666 adults [154], our pooled scores were lower in all four HRQOL domains. This may be due to the difference between our study and the two surveys in sampling strategies. The two surveys targeted general adults which may have some systematic difference in occupation distribution. However, this difference could not be verified because these two surveys did not report data on occupation composition. The publications we included were presenting vulnerable occupational groups with impaired well-being, such as workers with heavy physical load, high work intensity and high psychological pressure.

Although subgroup analyses were conducted to explore the source of heterogeneity, no statistical differences were found among gender, age, and occupation groups. Different results across regions might be due to the differences in population distribution and resource allocation. Central China, including Hubei, Hunan, and Henan province, is a densely populated area, thus per capita resources in transportation, living conditions and medical services are relatively less. However, the influence of differences in occupation distribution cannot be excluded.

All the above three occupational groups have their own specific occupational risk factors associated with poor HRQOL. Previous studies have shown that bluecollar workers often have harsh working conditions including ergonomic, environmental and psychological hazards. For example, heavy physical load, awkward working postures, vibration, extreme temperatures, noise, harmful chemicals were correlated with musculoskeletal disorders, heat-related illness, skin and lung diseases, and can lead to poor physical health [155–158]. A higher incidence of non-fatal work injuries and fatalities has also been seen among blue-collar workers, especially construction workers. From 2014 to 2018, 3024 municipal work accidents were reported in China, resulting in an average of 717 deaths per year in China’s construction industry [159]. Moreover, supervisor and coworkers support in the work environment were found to be essential predictors of the psychological health, social relationship and environmental domains of HRQOL [160]. For office workers, lack of ergonomic-featured office equipment, sitting, standing and watching computer screens for a long period, and lack of exercise, were related to arm, neck, shoulder and lower limbs pains as well as eye problems [161–163]. In addition, due to the low requirements for physical burden, office workers’ on-boarding health screening may be not as strict as blue-collar workers, and were less likely to quit work because of acute injuries. For health care workers, increased number of hospital visits by an aging population, strained doctor-patient relationships, and poor sleep habits are important detrimental factors for physical and psychological health, which can lead to occupational stress, depression, burnout and physical exhaustion [144].

Sex work is illegal and not considered as an occupation in China, therefore related publications were not included in our study. Jiang et al. found that female sex workers reported lower scores than women in general in the social relationship and environment domain, which was ascribed to high population mobility and lack of occupational safety and health services. Wang et al. [164] reported lower scores for sex workers than for the general population in the physical domain, which might be related to multiple sexual partners.

As a health indicator, the assessment of quality of life makes it possible to prospectively study of diseases. Our study summarizes overall HRQOL levels among Chinese occupational groups and provided a potential reference for future study. Based on our study, it appears that there remains a need to strengthen the occupational safety and health management of vulnerable occupational groups and reduce exposure to known health risk factors in the future. Government departments also need to rationally allocate resources such as medical care, housing conditions and transportation according to regional factors like economic development level, industrial distribution and employment status, etc. However, close observing the trend of HRQOL over time and identifying essential contributors in the next step are imperative for relevant policy planning.

The results of our study may be biased. The study quality of the included publications was often not satisfactory because of improper sampling methods and unverified reliability and validity. Besides, about half of the included publications focused on medical staff, thus the pooled scores might be close to their results. There are also some publications that reported the results of subgroups (such as migrant workers and urban workers) rather than the entire study population, and the combination of data may induce bias. In Chinese culture, endurance was considered as a merit and people tend to underreport their discomfort. In addition, people also prefer to choose medium instead of extreme figures, which may result in similar results.

There are several limitations to the study. First, the absence of blinding (author and publication information disclosed) used in the search and selection of publications may have leaded to researcher bias. Second, although the search strategy was comprehensive, there may still have been additional studies not indexed by the selected database. Third, given the difficulties in comparing results based on different HRQOL instruments, our systematic review excluded studies that used other instruments (such as 36-Item Short-Form Health Survey, the symptom checklist-90) than WHOQOL-100 or WHOQOL-BREF. Fourth, of the included publications, some did not report the average age, gender and occupation of the participants, which may represent a group of workers with distinct results and lead to bias for subgroup analysis.

## Conclusion

This is the first systematic review to synthesize the HRQOL scores for Chinese workers. The pooled scores in HRQOL were lower than those in the general population. Subgroup analysis did not suggest a strong relationship of gender, age and types of job with HRQOL scores, and region might be a source of heterogeneity. We suggest that future HRQOL studies pay more attention to these factors so that effective occupational safety and health targeted to specific groups can be developed and implemented.

## Abbreviations

HRQOL: Health-related quality of life
WHOQOL: The World Health Organization Quality of Life
CNKI: China National Knowledge Infrastructure
WF: WanFang Data
CQVIP: China Science and Technology Journal Database
MeSH: Medical subject heading
PRISMA: Preferred reporting items for systematic reviews and meta-analyses
WHO: World Health Organization
