# Correction to: Health-related quality of life in Chinese workers: a systematic review and meta-analysis

**DOI:** 10.1186/s41256-021-00218-y

**Published:** 2021-09-03

**Authors:** Ya Su, Meng-Shuang Liu, Pinnaduwage Vijitha De Silva, Truls Østbye, Ke-Zhi Jin

**Affiliations:** 1 Department of Occupational Health, School of Public Health, Fudan University, 138 Yixueyuan Road Box288#, Shanghai, 200032 People’s Republic of China; 2 Key Laboratory of Public Health Safety, Ministry of Education, Shanghai, People’s Republic of China; 3 Fudan Global Health Institute, Fudan University, Shanghai, People’s Republic of China; 4 Department of Community Medicine, University of Ruhuna, Galle, Sri Lanka; 5 Duke Global Health Institute, Durham, NC USA

## Correction to: Global Health Research and Policy (2021) 6:29 https://doi.org/10.1186/s41256-021-00209-z

Following publication of the original article [1], it is reported that there were errors in the first author’s name and the third affiliation.

The incorrect author's name is "By Ya Su".

The correct author name is "Ya Su".

The incorrect affiliation is:

^3^Fudan Global Health Institute, Shanghai, People’s Republic of China.

The correct affiliation is:

^3^Fudan Global Health Institute, Fudan University, Shanghai, People’s Republic of China.

The first author name and the third affiliation have been corrected above and the original article [1] has been updated as well.

## References

[1] Su Y, Liu MS, De Silva PV (2021). Health-related quality of life in Chinese workers: a systematic review and meta-analysis. Glob health Res policy.

